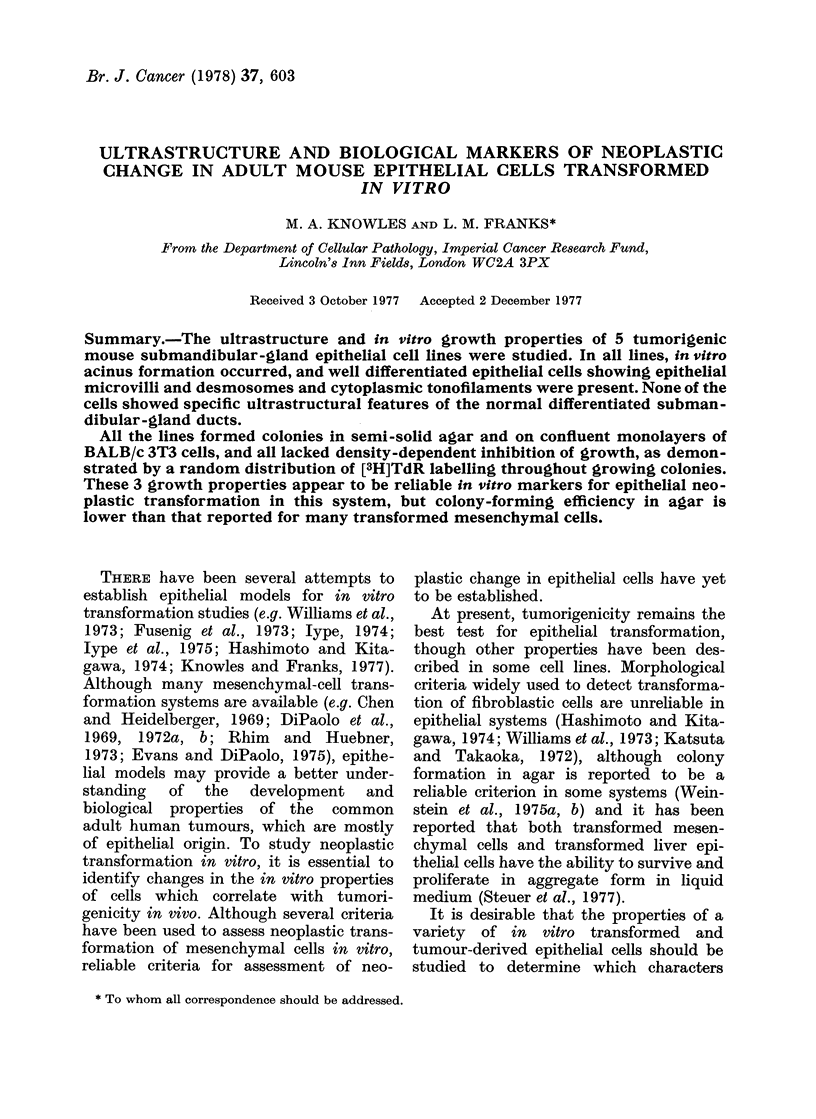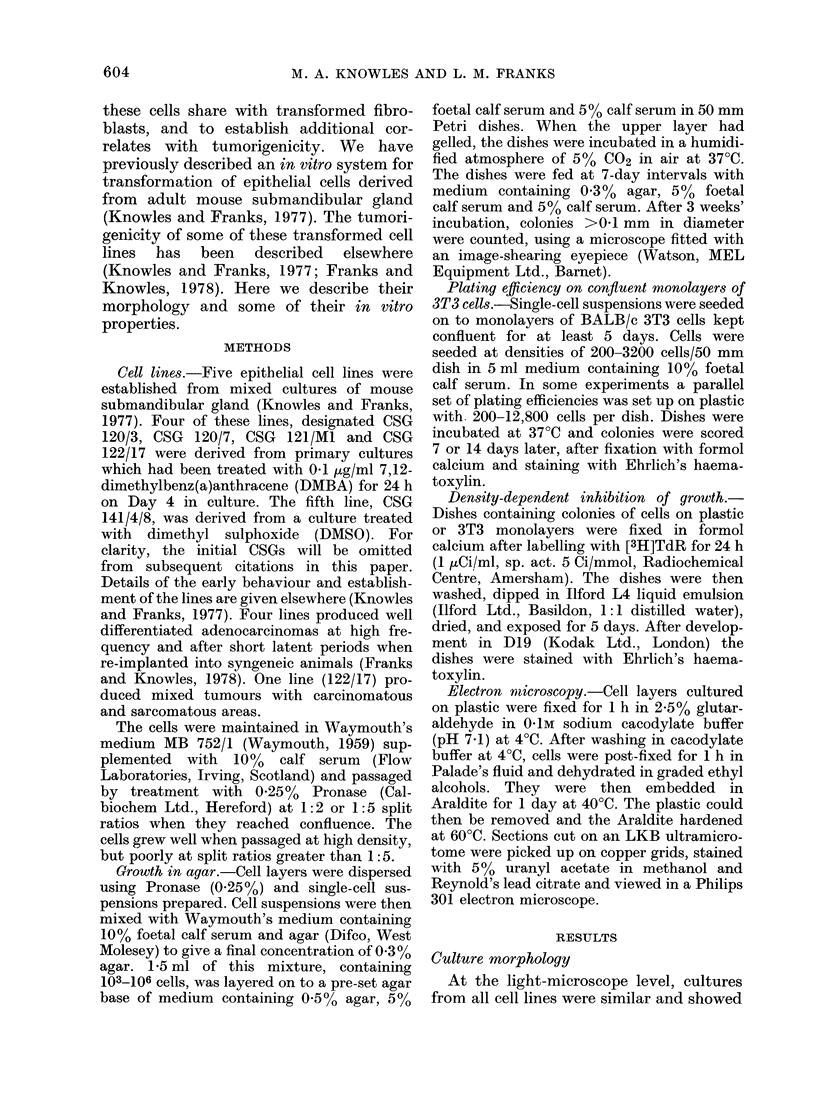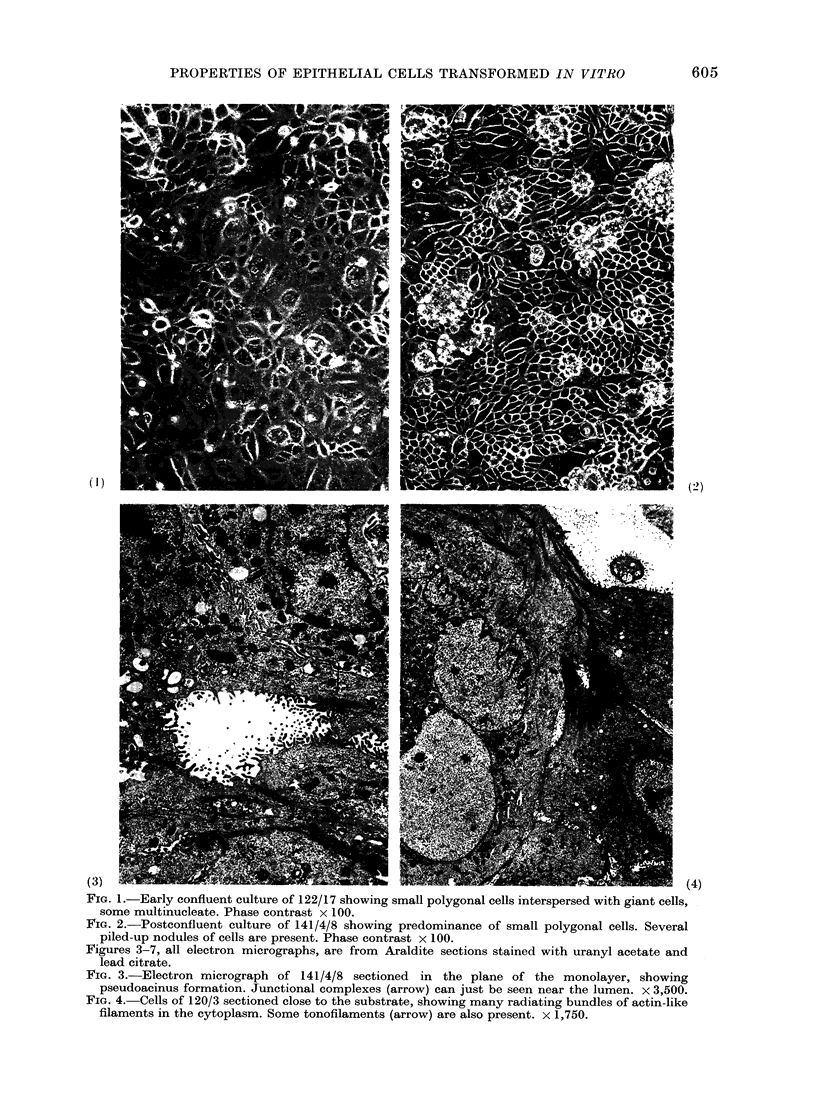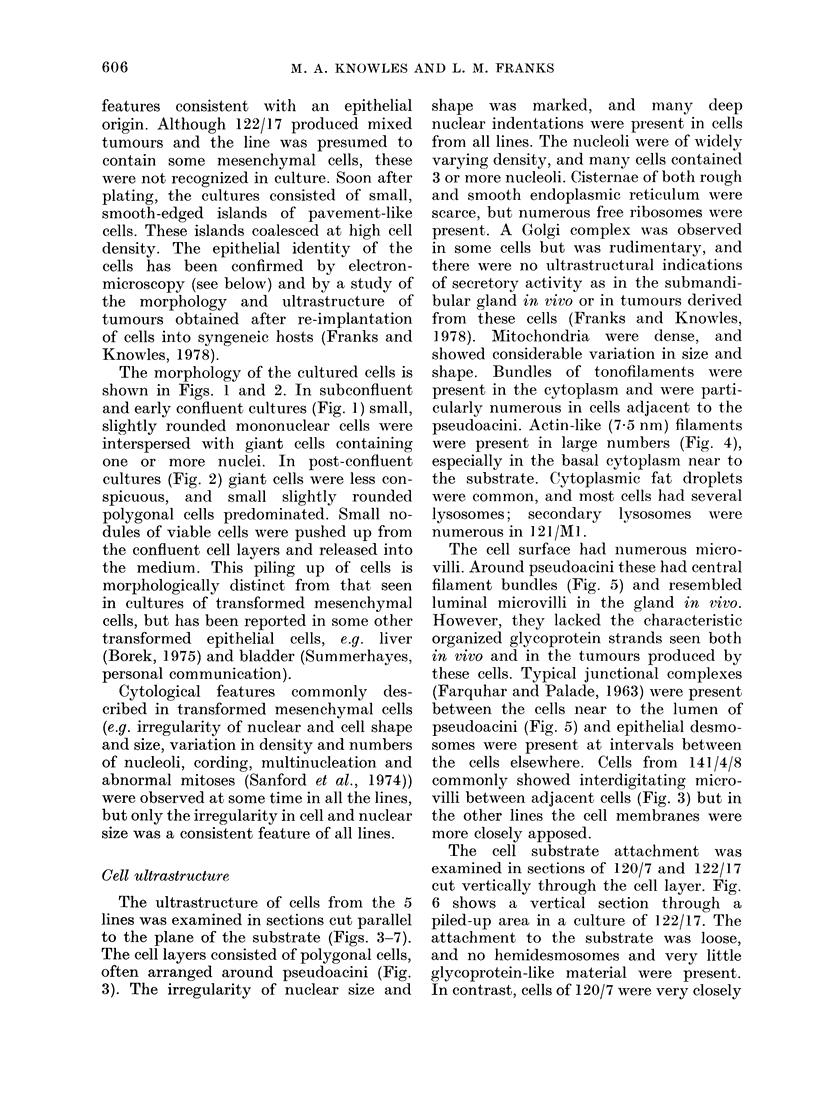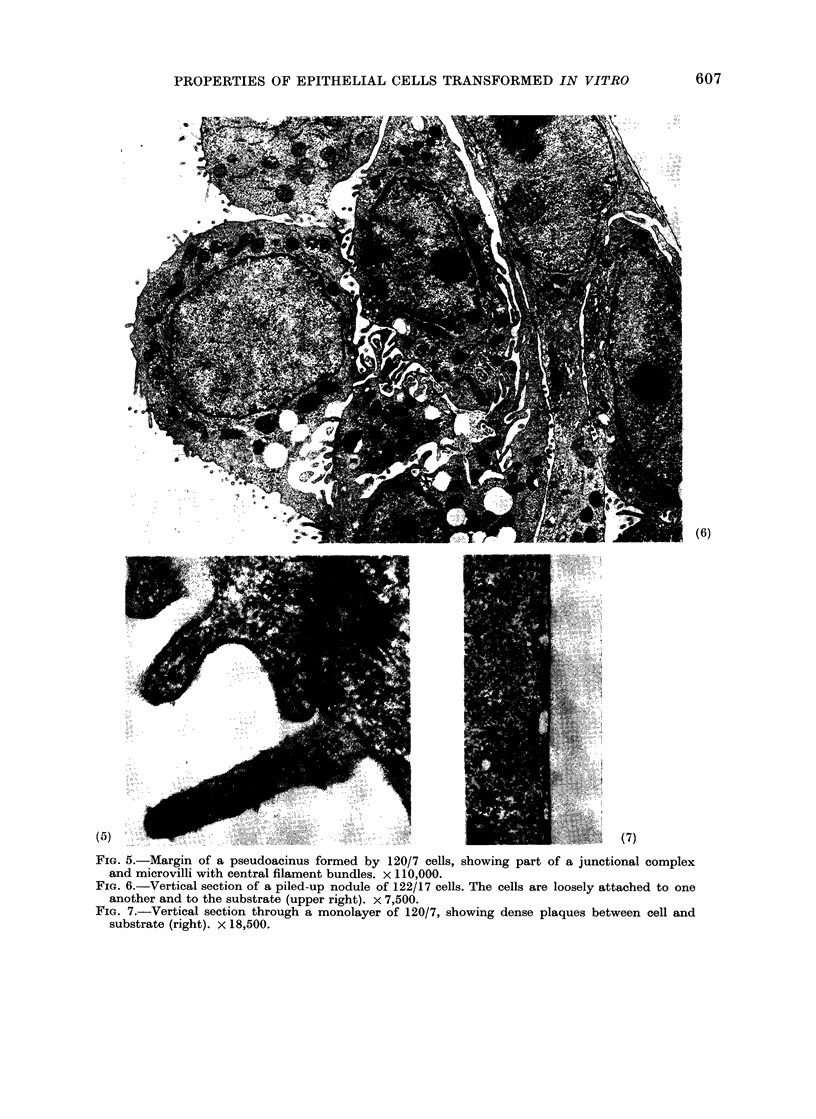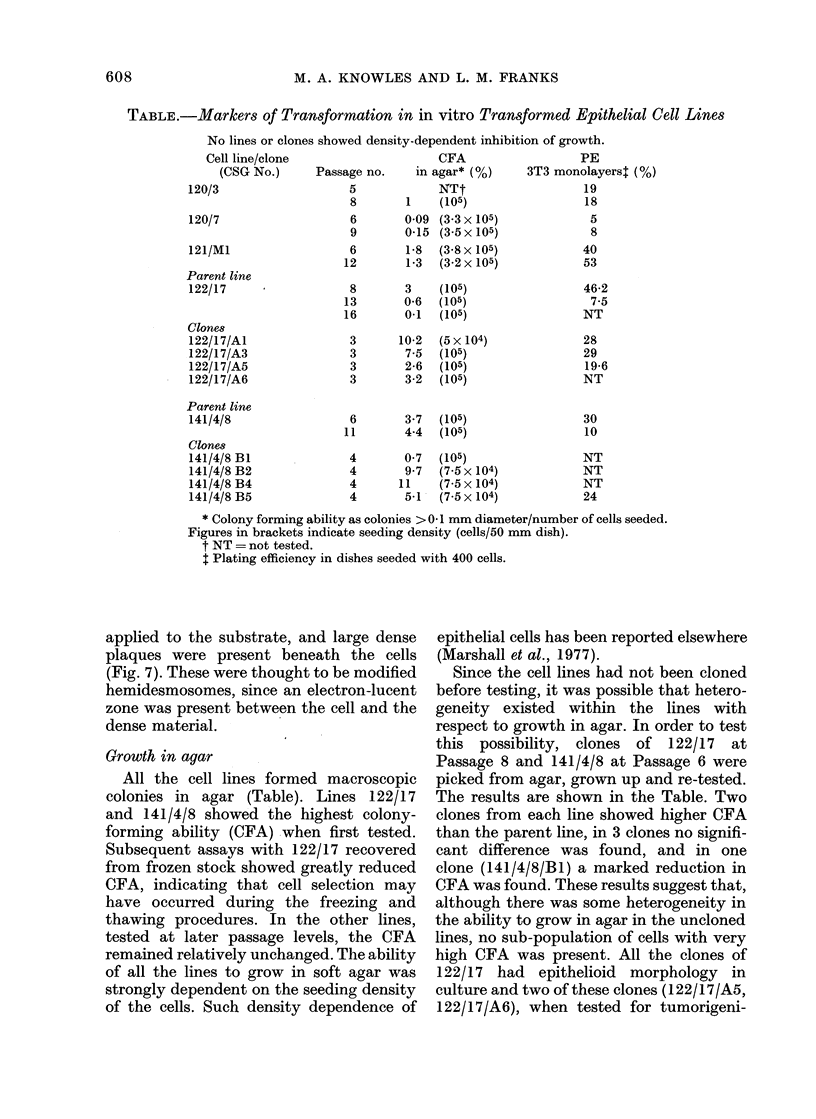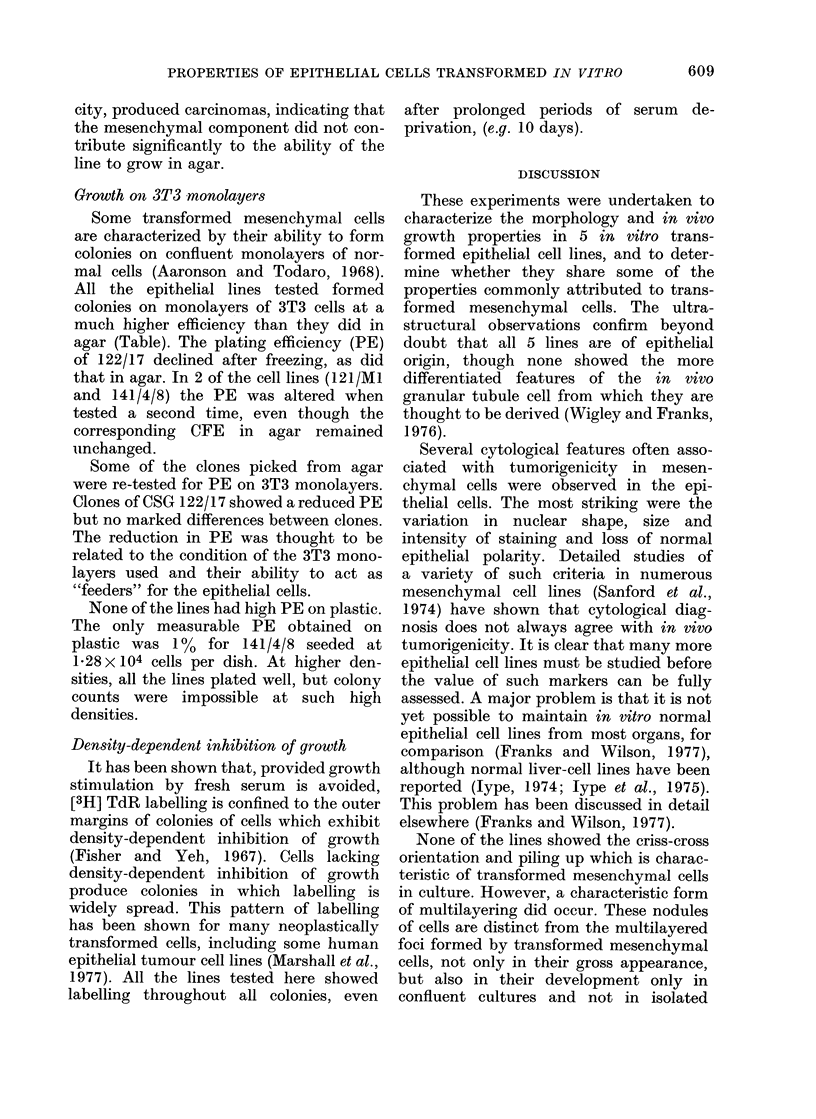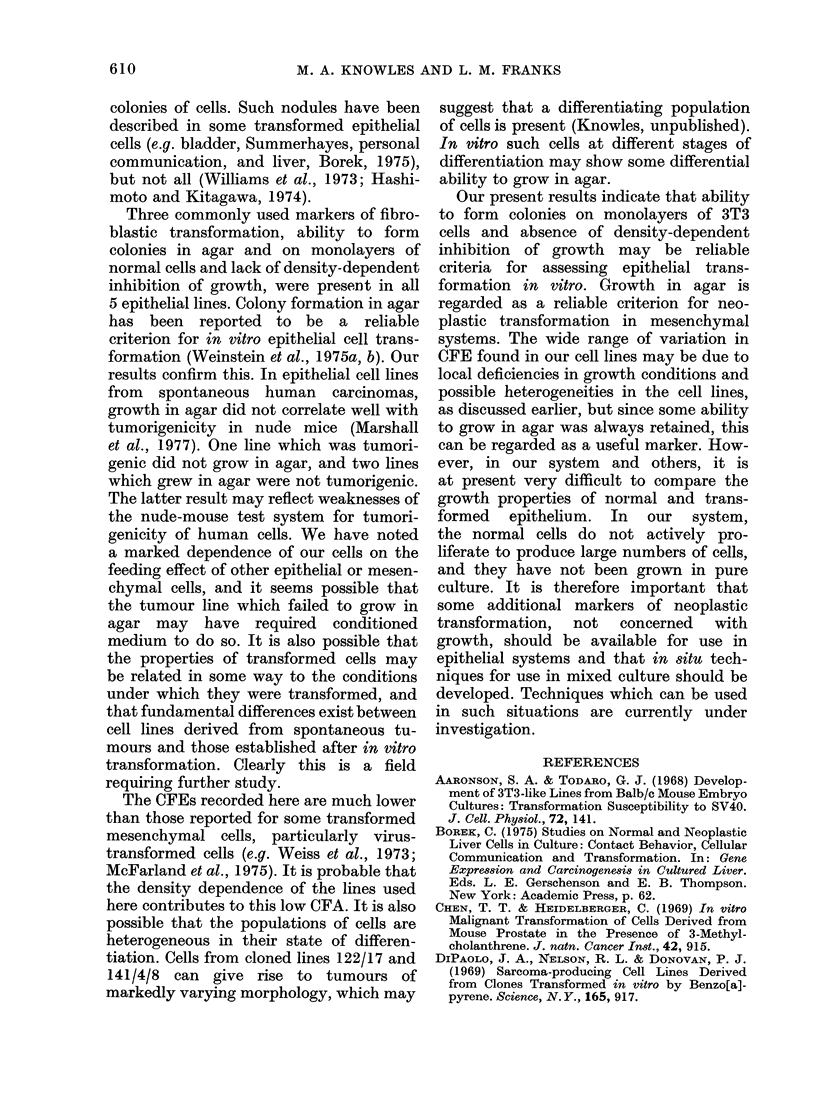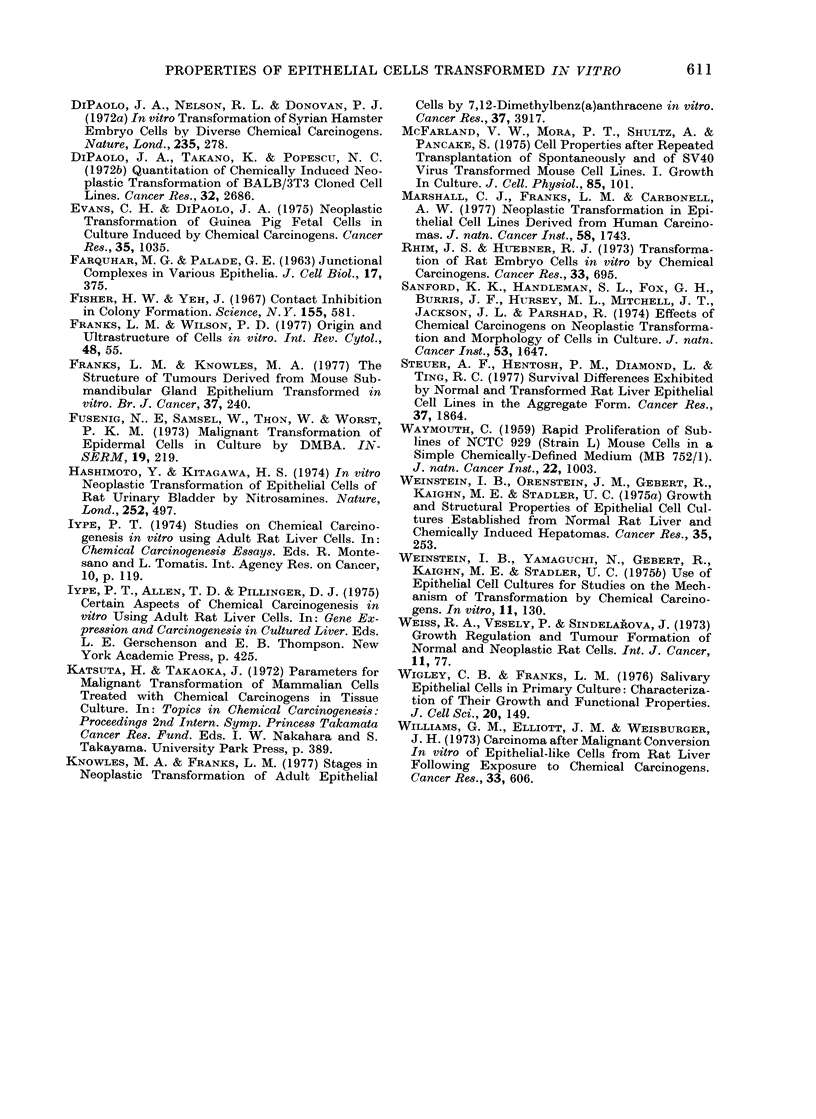# Ultrastructure and biological markers of neoplastic change in adult mouse epithelial cells transformed in vitro.

**DOI:** 10.1038/bjc.1978.90

**Published:** 1978-04

**Authors:** M. A. Knowles, L. M. Franks

## Abstract

**Images:**


*Br. J. Cancer* (1978) **37**, 603

### ULTRASTRUCTURE AND BIOLOGICAL MARKERS OF NEOPLASTIC CHANGE IN ADULT MOUSE EPITHELIAL CELLS TRANSFORMED *IN VITRO*

M. A. KNOWLES AND L. M. FRANKS*

*From the Department of Cellular Pathology, Imperial Cancer Research Fund, Lincoln's Inn Fields, London WC2A 3PX*

Received 3 October 1977 Accepted 2 December 1977

**Summary.—The ultrastructure and *in vitro* growth properties of 5 tumorigenic mouse submandibular-gland epithelial cell lines were studied. In all lines, *in vitro* acinus formation occurred, and well differentiated epithelial cells showing epithelial microvilli and desmosomes and cytoplasmic tonofilaments were present. None of the cells showed specific ultrastructural features of the normal differentiated submandibular-gland ducts.**

**All the lines formed colonies in semi-solid agar and on confluent monolayers of BALB/c 3T3 cells, and all lacked density-dependent inhibition of growth, as demonstrated by a random distribution of [$^3$H]TdR labelling throughout growing colonies. These 3 growth properties appear to be reliable *in vitro* markers for epithelial neoplastic transformation in this system, but colony-forming efficiency in agar is lower than that reported for many transformed mesenchymal cells.**

THERE have been several attempts to establish epithelial models for *in vitro* transformation studies (*e.g.* Williams *et al.*, 1973; Fusenig *et al.*, 1973; Iype, 1974; Iype *et al.*, 1975; Hashimoto and Kitagawa, 1974; Knowles and Franks, 1977). Although many mesenchymal-cell transformation systems are available (*e.g.* Chen and Heidelberger, 1969; DiPaolo *et al.*, 1969, 1972*a*, *b*; Rhim and Huebner, 1973; Evans and DiPaolo, 1975), epithelial models may provide a better understanding of the development and biological properties of the common adult human tumours, which are mostly of epithelial origin. To study neoplastic transformation *in vitro*, it is essential to identify changes in the *in vitro* properties of cells which correlate with tumorigenicity *in vivo*. Although several criteria have been used to assess neoplastic transformation of mesenchymal cells *in vitro*, reliable criteria for assessment of neoplastic change in epithelial cells have yet to be established.

At present, tumorigenicity remains the best test for epithelial transformation, though other properties have been described in some cell lines. Morphological criteria widely used to detect transformation of fibroblastic cells are unreliable in epithelial systems (Hashimoto and Kitagawa, 1974; Williams *et al.*, 1973; Katsuta and Takaoka, 1972), although colony formation in agar is reported to be a reliable criterion in some systems (Weinstein *et al.*, 1975*a*, *b*) and it has been reported that both transformed mesenchymal cells and transformed liver epithelial cells have the ability to survive and proliferate in aggregate form in liquid medium (Steuer *et al.*, 1977).

It is desirable that the properties of a variety of *in vitro* transformed and tumour-derived epithelial cells should be studied to determine which characters

* To whom all correspondence should be addressed.

these cells share with transformed fibroblasts, and to establish additional correlates with tumorigenicity. We have previously described an *in vitro* system for transformation of epithelial cells derived from adult mouse submandibular gland (Knowles and Franks, 1977). The tumorigenicity of some of these transformed cell lines has been described elsewhere (Knowles and Franks, 1977; Franks and Knowles, 1978). Here we describe their morphology and some of their *in vitro* properties.

### METHODS

*Cell lines.*—Five epithelial cell lines were established from mixed cultures of mouse submandibular gland (Knowles and Franks, 1977). Four of these lines, designated CSG 120/3, CSG 120/7, CSG 121/M1 and CSG 122/17 were derived from primary cultures which had been treated with 0·1 $\mu$g/ml 7,12-dimethylbenz(a)anthracene (DMBA) for 24 h on Day 4 in culture. The fifth line, CSG 141/4/8, was derived from a culture treated with dimethyl sulphoxide (DMSO). For clarity, the initial CSGs will be omitted from subsequent citations in this paper. Details of the early behaviour and establishment of the lines are given elsewhere (Knowles and Franks, 1977). Four lines produced well differentiated adenocarcinomas at high frequency and after short latent periods when re-implanted into syngeneic animals (Franks and Knowles, 1978). One line (122/17) produced mixed tumours with carcinomatous and sarcomatous areas.

The cells were maintained in Waymouth's medium MB 752/1 (Waymouth, 1959) supplemented with 10% calf serum (Flow Laboratories, Irving, Scotland) and passaged by treatment with 0·25% Pronase (Calbiochem Ltd., Hereford) at 1:2 or 1:5 split ratios when they reached confluence. The cells grew well when passaged at high density, but poorly at split ratios greater than 1:5.

*Growth in agar.*—Cell layers were dispersed using Pronase (0·25%) and single-cell suspensions prepared. Cell suspensions were then mixed with Waymouth's medium containing 10% foetal calf serum and agar (Difco, West Molesey) to give a final concentration of 0·3% agar. 1·5 ml of this mixture, containing $10^3$–$10^6$ cells, was layered on to a pre-set agar base of medium containing 0·5% agar, 5% foetal calf serum and 5% calf serum in 50 mm Petri dishes. When the upper layer had gelled, the dishes were incubated in a humidified atmosphere of 5% $CO_2$ in air at 37°C. The dishes were fed at 7-day intervals with medium containing 0·3% agar, 5% foetal calf serum and 5% calf serum. After 3 weeks' incubation, colonies >0·1 mm in diameter were counted, using a microscope fitted with an image-shearing eyepiece (Watson, MEL Equipment Ltd., Barnet).

*Plating efficiency on confluent monolayers of 3T3 cells.*—Single-cell suspensions were seeded on to monolayers of BALB/c 3T3 cells kept confluent for at least 5 days. Cells were seeded at densities of 200–3200 cells/50 mm dish in 5 ml medium containing 10% foetal calf serum. In some experiments a parallel set of plating efficiencies was set up on plastic with 200–12,800 cells per dish. Dishes were incubated at 37°C and colonies were scored 7 or 14 days later, after fixation with formol calcium and staining with Ehrlich's haematoxylin.

*Density-dependent inhibition of growth.*—Dishes containing colonies of cells on plastic or 3T3 monolayers were fixed in formol calcium after labelling with [$^3$H]TdR for 24 h (1 $\mu$Ci/ml, sp. act. 5 Ci/mmol, Radiochemical Centre, Amersham). The dishes were then washed, dipped in Ilford L4 liquid emulsion (Ilford Ltd., Basildon, 1:1 distilled water), dried, and exposed for 5 days. After development in D19 (Kodak Ltd., London) the dishes were stained with Ehrlich's haematoxylin.

*Electron microscopy.*—Cell layers cultured on plastic were fixed for 1 h in 2·5% glutaraldehyde in 0·1M sodium cacodylate buffer (pH 7·1) at 4°C. After washing in cacodylate buffer at 4°C, cells were post-fixed for 1 h in Palade's fluid and dehydrated in graded ethyl alcohols. They were then embedded in Araldite for 1 day at 40°C. The plastic could then be removed and the Araldite hardened at 60°C. Sections cut on an LKB ultramicrotome were picked up on copper grids, stained with 5% uranyl acetate in methanol and Reynold's lead citrate and viewed in a Philips 301 electron microscope.

### RESULTS

### *Culture morphology*

At the light-microscope level, cultures from all cell lines were similar and showed

(1)

(2)

(3)

(4)

FIG. 1.—Early confluent culture of 122/17 showing small polygonal cells interspersed with giant cells, some multinucleate. Phase contrast ×100.

FIG. 2.—Postconfluent culture of 141/4/8 showing predominance of small polygonal cells. Several piled-up nodules of cells are present. Phase contrast ×100.

Figures 3–7, all electron micrographs, are from Araldite sections stained with uranyl acetate and lead citrate.

FIG. 3.—Electron micrograph of 141/4/8 sectioned in the plane of the monolayer, showing pseudoacinus formation. Junctional complexes (arrow) can just be seen near the lumen. ×3,500.

FIG. 4.—Cells of 120/3 sectioned close to the substrate, showing many radiating bundles of actin-like filaments in the cytoplasm. Some tonofilaments (arrow) are also present. ×1,750.

features consistent with an epithelial origin. Although 122/17 produced mixed tumours and the line was presumed to contain some mesenchymal cells, these were not recognized in culture. Soon after plating, the cultures consisted of small, smooth-edged islands of pavement-like cells. These islands coalesced at high cell density. The epithelial identity of the cells has been confirmed by electron-microscopy (see below) and by a study of the morphology and ultrastructure of tumours obtained after re-implantation of cells into syngeneic hosts (Franks and Knowles, 1978).

The morphology of the cultured cells is shown in Figs. 1 and 2. In subconfluent and early confluent cultures (Fig. 1) small, slightly rounded mononuclear cells were interspersed with giant cells containing one or more nuclei. In post-confluent cultures (Fig. 2) giant cells were less conspicuous, and small slightly rounded polygonal cells predominated. Small nodules of viable cells were pushed up from the confluent cell layers and released into the medium. This piling up of cells is morphologically distinct from that seen in cultures of transformed mesenchymal cells, but has been reported in some other transformed epithelial cells, *e.g.* liver (Borek, 1975) and bladder (Summerhayes, personal communication).

Cytological features commonly described in transformed mesenchymal cells (*e.g.* irregularity of nuclear and cell shape and size, variation in density and numbers of nucleoli, cording, multinucleation and abnormal mitoses (Sanford *et al.*, 1974)) were observed at some time in all the lines, but only the irregularity in cell and nuclear size was a consistent feature of all lines.

*Cell ultrastructure*

The ultrastructure of cells from the 5 lines was examined in sections cut parallel to the plane of the substrate (Figs. 3–7). The cell layers consisted of polygonal cells, often arranged around pseudoacini (Fig. 3). The irregularity of nuclear size and shape was marked, and many deep nuclear indentations were present in cells from all lines. The nucleoli were of widely varying density, and many cells contained 3 or more nucleoli. Cisternae of both rough and smooth endoplasmic reticulum were scarce, but numerous free ribosomes were present. A Golgi complex was observed in some cells but was rudimentary, and there were no ultrastructural indications of secretory activity as in the submandibular gland *in vivo* or in tumours derived from these cells (Franks and Knowles, 1978). Mitochondria were dense, and showed considerable variation in size and shape. Bundles of tonofilaments were present in the cytoplasm and were particularly numerous in cells adjacent to the pseudoacini. Actin-like (7·5 nm) filaments were present in large numbers (Fig. 4), especially in the basal cytoplasm near to the substrate. Cytoplasmic fat droplets were common, and most cells had several lysosomes; secondary lysosomes were numerous in 121/M1.

The cell surface had numerous microvilli. Around pseudoacini these had central filament bundles (Fig. 5) and resembled luminal microvilli in the gland *in vivo*. However, they lacked the characteristic organized glycoprotein strands seen both *in vivo* and in the tumours produced by these cells. Typical junctional complexes (Farquhar and Palade, 1963) were present between the cells near to the lumen of pseudoacini (Fig. 5) and epithelial desmosomes were present at intervals between the cells elsewhere. Cells from 141/4/8 commonly showed interdigitating microvilli between adjacent cells (Fig. 3) but in the other lines the cell membranes were more closely apposed.

The cell substrate attachment was examined in sections of 120/7 and 122/17 cut vertically through the cell layer. Fig. 6 shows a vertical section through a piled-up area in a culture of 122/17. The attachment to the substrate was loose, and no hemidesmosomes and very little glycoprotein-like material were present. In contrast, cells of 120/7 were very closely

(6)

(5)

(7)

FIG. 5.—Margin of a pseudoacinus formed by 120/7 cells, showing part of a junctional complex and microvilli with central filament bundles. × 110,000.

FIG. 6.—Vertical section of a piled-up nodule of 122/17 cells. The cells are loosely attached to one another and to the substrate (upper right). × 7,500.

FIG. 7.—Vertical section through a monolayer of 120/7, showing dense plaques between cell and substrate (right). × 18,500.

TABLE.—*Markers of Transformation in* in vitro *Transformed Epithelial Cell Lines*

No lines or clones showed density-dependent inhibition of growth.

| Cell line/clone (CSG No.) | Passage no. | CFA in agar* (%) | PE 3T3 monolayers‡ (%) |
|---|---|---|---|
| 120/3 | 5 | NT† | 19 |
| | 8 | 1 $(10^5)$ | 18 |
| 120/7 | 6 | 0·09 $(3{\cdot}3\times10^5)$ | 5 |
| | 9 | 0·15 $(3{\cdot}5\times10^5)$ | 8 |
| 121/M1 | 6 | 1·8 $(3{\cdot}8\times10^5)$ | 40 |
| | 12 | 1·3 $(3{\cdot}2\times10^5)$ | 53 |
| *Parent line* | | | |
| 122/17 | 8 | 3 $(10^5)$ | 46·2 |
| | 13 | 0·6 $(10^5)$ | 7·5 |
| | 16 | 0·1 $(10^5)$ | NT |
| *Clones* | | | |
| 122/17/A1 | 3 | 10·2 $(5\times10^4)$ | 28 |
| 122/17/A3 | 3 | 7·5 $(10^5)$ | 29 |
| 122/17/A5 | 3 | 2·6 $(10^5)$ | 19·6 |
| 122/17/A6 | 3 | 3·2 $(10^5)$ | NT |
| *Parent line* | | | |
| 141/4/8 | 6 | 3·7 $(10^5)$ | 30 |
| | 11 | 4·4 $(10^5)$ | 10 |
| *Clones* | | | |
| 141/4/8 B1 | 4 | 0·7 $(10^5)$ | NT |
| 141/4/8 B2 | 4 | 9·7 $(7{\cdot}5\times10^4)$ | NT |
| 141/4/8 B4 | 4 | 11 $(7{\cdot}5\times10^4)$ | NT |
| 141/4/8 B5 | 4 | 5·1 $(7{\cdot}5\times10^4)$ | 24 |

* Colony forming ability as colonies $>0{\cdot}1$ mm diameter/number of cells seeded. Figures in brackets indicate seeding density (cells/50 mm dish).
† NT = not tested.
‡ Plating efficiency in dishes seeded with 400 cells.

applied to the substrate, and large dense plaques were present beneath the cells (Fig. 7). These were thought to be modified hemidesmosomes, since an electron-lucent zone was present between the cell and the dense material.

*Growth in agar*

All the cell lines formed macroscopic colonies in agar (Table). Lines 122/17 and 141/4/8 showed the highest colony-forming ability (CFA) when first tested. Subsequent assays with 122/17 recovered from frozen stock showed greatly reduced CFA, indicating that cell selection may have occurred during the freezing and thawing procedures. In the other lines, tested at later passage levels, the CFA remained relatively unchanged. The ability of all the lines to grow in soft agar was strongly dependent on the seeding density of the cells. Such density dependence of epithelial cells has been reported elsewhere (Marshall *et al.*, 1977).

Since the cell lines had not been cloned before testing, it was possible that heterogeneity existed within the lines with respect to growth in agar. In order to test this possibility, clones of 122/17 at Passage 8 and 141/4/8 at Passage 6 were picked from agar, grown up and re-tested. The results are shown in the Table. Two clones from each line showed higher CFA than the parent line, in 3 clones no significant difference was found, and in one clone (141/4/8/B1) a marked reduction in CFA was found. These results suggest that, although there was some heterogeneity in the ability to grow in agar in the uncloned lines, no sub-population of cells with very high CFA was present. All the clones of 122/17 had epithelioid morphology in culture and two of these clones (122/17/A5, 122/17/A6), when tested for tumorigenicity, produced carcinomas, indicating that the mesenchymal component did not contribute significantly to the ability of the line to grow in agar.

*Growth on 3T3 monolayers*

Some transformed mesenchymal cells are characterized by their ability to form colonies on confluent monolayers of normal cells (Aaronson and Todaro, 1968). All the epithelial lines tested formed colonies on monolayers of 3T3 cells at a much higher efficiency than they did in agar (Table). The plating efficiency (PE) of 122/17 declined after freezing, as did that in agar. In 2 of the cell lines (121/M1 and 141/4/8) the PE was altered when tested a second time, even though the corresponding CFE in agar remained unchanged.

Some of the clones picked from agar were re-tested for PE on 3T3 monolayers. Clones of CSG 122/17 showed a reduced PE but no marked differences between clones. The reduction in PE was thought to be related to the condition of the 3T3 monolayers used and their ability to act as "feeders" for the epithelial cells.

None of the lines had high PE on plastic. The only measurable PE obtained on plastic was 1% for 141/4/8 seeded at $1{\cdot}28 \times 10^4$ cells per dish. At higher densities, all the lines plated well, but colony counts were impossible at such high densities.

*Density-dependent inhibition of growth*

It has been shown that, provided growth stimulation by fresh serum is avoided, [$^3$H] TdR labelling is confined to the outer margins of colonies of cells which exhibit density-dependent inhibition of growth (Fisher and Yeh, 1967). Cells lacking density-dependent inhibition of growth produce colonies in which labelling is widely spread. This pattern of labelling has been shown for many neoplastically transformed cells, including some human epithelial tumour cell lines (Marshall *et al.*, 1977). All the lines tested here showed labelling throughout all colonies, even after prolonged periods of serum deprivation, (*e.g.* 10 days).

DISCUSSION

These experiments were undertaken to characterize the morphology and *in vivo* growth properties in 5 *in vitro* transformed epithelial cell lines, and to determine whether they share some of the properties commonly attributed to transformed mesenchymal cells. The ultrastructural observations confirm beyond doubt that all 5 lines are of epithelial origin, though none showed the more differentiated features of the *in vivo* granular tubule cell from which they are thought to be derived (Wigley and Franks, 1976).

Several cytological features often associated with tumorigenicity in mesenchymal cells were observed in the epithelial cells. The most striking were the variation in nuclear shape, size and intensity of staining and loss of normal epithelial polarity. Detailed studies of a variety of such criteria in numerous mesenchymal cell lines (Sanford *et al.*, 1974) have shown that cytological diagnosis does not always agree with *in vivo* tumorigenicity. It is clear that many more epithelial cell lines must be studied before the value of such markers can be fully assessed. A major problem is that it is not yet possible to maintain *in vitro* normal epithelial cell lines from most organs, for comparison (Franks and Wilson, 1977), although normal liver-cell lines have been reported (Iype, 1974; Iype *et al.*, 1975). This problem has been discussed in detail elsewhere (Franks and Wilson, 1977).

None of the lines showed the criss-cross orientation and piling up which is characteristic of transformed mesenchymal cells in culture. However, a characteristic form of multilayering did occur. These nodules of cells are distinct from the multilayered foci formed by transformed mesenchymal cells, not only in their gross appearance, but also in their development only in confluent cultures and not in isolated colonies of cells. Such nodules have been described in some transformed epithelial cells (*e.g.* bladder, Summerhayes, personal communication, and liver, Borek, 1975), but not all (Williams *et al.*, 1973; Hashimoto and Kitagawa, 1974).

Three commonly used markers of fibroblastic transformation, ability to form colonies in agar and on monolayers of normal cells and lack of density-dependent inhibition of growth, were present in all 5 epithelial lines. Colony formation in agar has been reported to be a reliable criterion for *in vitro* epithelial cell transformation (Weinstein *et al.*, 1975*a*, *b*). Our results confirm this. In epithelial cell lines from spontaneous human carcinomas, growth in agar did not correlate well with tumorigenicity in nude mice (Marshall *et al.*, 1977). One line which was tumorigenic did not grow in agar, and two lines which grew in agar were not tumorigenic. The latter result may reflect weaknesses of the nude-mouse test system for tumorigenicity of human cells. We have noted a marked dependence of our cells on the feeding effect of other epithelial or mesenchymal cells, and it seems possible that the tumour line which failed to grow in agar may have required conditioned medium to do so. It is also possible that the properties of transformed cells may be related in some way to the conditions under which they were transformed, and that fundamental differences exist between cell lines derived from spontaneous tumours and those established after *in vitro* transformation. Clearly this is a field requiring further study.

The CFEs recorded here are much lower than those reported for some transformed mesenchymal cells, particularly virus-transformed cells (*e.g.* Weiss *et al.*, 1973; McFarland *et al.*, 1975). It is probable that the density dependence of the lines used here contributes to this low CFA. It is also possible that the populations of cells are heterogeneous in their state of differentiation. Cells from cloned lines 122/17 and 141/4/8 can give rise to tumours of markedly varying morphology, which may suggest that a differentiating population of cells is present (Knowles, unpublished). *In vitro* such cells at different stages of differentiation may show some differential ability to grow in agar.

Our present results indicate that ability to form colonies on monolayers of 3T3 cells and absence of density-dependent inhibition of growth may be reliable criteria for assessing epithelial transformation *in vitro*. Growth in agar is regarded as a reliable criterion for neoplastic transformation in mesenchymal systems. The wide range of variation in CFE found in our cell lines may be due to local deficiencies in growth conditions and possible heterogeneities in the cell lines, as discussed earlier, but since some ability to grow in agar was always retained, this can be regarded as a useful marker. However, in our system and others, it is at present very difficult to compare the growth properties of normal and transformed epithelium. In our system, the normal cells do not actively proliferate to produce large numbers of cells, and they have not been grown in pure culture. It is therefore important that some additional markers of neoplastic transformation, not concerned with growth, should be available for use in epithelial systems and that *in situ* techniques for use in mixed culture should be developed. Techniques which can be used in such situations are currently under investigation.

### REFERENCES

AARONSON, S. A. & TODARO, G. J. (1968) Development of 3T3-like Lines from Balb/c Mouse Embryo Cultures: Transformation Susceptibility to SV40. *J. Cell. Physiol.*, **72**, 141.

BOREK, C. (1975) Studies on Normal and Neoplastic Liver Cells in Culture: Contact Behavior, Cellular Communication and Transformation. In: *Gene Expression and Carcinogenesis in Cultured Liver.* Eds. L. E. Gerschenson and E. B. Thompson. New York: Academic Press, p. 62.

CHEN, T. T. & HEIDELBERGER, C. (1969) *In vitro* Malignant Transformation of Cells Derived from Mouse Prostate in the Presence of 3-Methylcholanthrene. *J. natn. Cancer Inst.*, **42**, 915.

DIPAOLO, J. A., NELSON, R. L. & DONOVAN, P. J. (1969) Sarcoma-producing Cell Lines Derived from Clones Transformed *in vitro* by Benzo[a]-pyrene. *Science, N.Y.*, **165**, 917.

DIPAOLO, J. A., NELSON, R. L. & DONOVAN, P. J. (1972*a*) *In vitro* Transformation of Syrian Hamster Embryo Cells by Diverse Chemical Carcinogens. *Nature, Lond.*, **235**, 278.

DIPAOLO, J. A., TAKANO, K. & POPESCU, N. C. (1972*b*) Quantitation of Chemically Induced Neoplastic Transformation of BALB/3T3 Cloned Cell Lines. *Cancer Res.*, **32**, 2686.

EVANS, C. H. & DIPAOLO, J. A. (1975) Neoplastic Transformation of Guinea Pig Fetal Cells in Culture Induced by Chemical Carcinogens. *Cancer Res.*, **35**, 1035.

FARQUHAR, M. G. & PALADE, G. E. (1963) Junctional Complexes in Various Epithelia. *J. Cell Biol.*, **17**, 375.

FISHER, H. W. & YEH, J. (1967) Contact Inhibition in Colony Formation. *Science, N.Y.* **155**, 581.

FRANKS, L. M. & WILSON, P. D. (1977) Origin and Ultrastructure of Cells *in vitro*. *Int. Rev. Cytol.*, **48**, 55.

FRANKS, L. M. & KNOWLES, M. A. (1977) The Structure of Tumours Derived from Mouse Submandibular Gland Epithelium Transformed *in vitro*. *Br. J. Cancer*, **37**, 240.

FUSENIG, N.. E, SAMSEL, W., THON, W. & WORST, P. K. M. (1973) Malignant Transformation of Epidermal Cells in Culture by DMBA. *INSERM*, **19**, 219.

HASHIMOTO, Y. & KITAGAWA, H. S. (1974) *In vitro* Neoplastic Transformation of Epithelial Cells of Rat Urinary Bladder by Nitrosamines. *Nature, Lond.*, **252**, 497.

IYPE, P. T. (1974) Studies on Chemical Carcinogenesis *in vitro* using Adult Rat Liver Cells. In: *Chemical Carcinogenesis Essays*. Eds. R. Montesano and L. Tomatis. Int. Agency Res. on Cancer, 10, p. 119.

IYPE, P. T., ALLEN, T. D. & PILLINGER, D. J. (1975) Certain Aspects of Chemical Carcinogenesis *in vitro* Using Adult Rat Liver Cells. In: *Gene Expression and Carcinogenesis in Cultured Liver*. Eds. L. E. Gerschenson and E. B. Thompson. New York Academic Press, p. 425.

KATSUTA, H. & TAKAOKA, J. (1972) Parameters for Malignant Transformation of Mammalian Cells Treated with Chemical Carcinogens in Tissue Culture. In: *Topics in Chemical Carcinogenesis: Proceedings 2nd Intern. Symp. Princess Takamata Cancer Res. Fund.* Eds. I. W. Nakahara and S. Takayama. University Park Press, p. 389.

KNOWLES, M. A. & FRANKS, L. M. (1977) Stages in Neoplastic Transformation of Adult Epithelial Cells by 7,12-Dimethylbenz(a)anthracene *in vitro*. *Cancer Res.*, **37**, 3917.

MCFARLAND, V. W., MORA, P. T., SHULTZ, A. & PANCAKE, S. (1975) Cell Properties after Repeated Transplantation of Spontaneously and of SV40 Virus Transformed Mouse Cell Lines. I. Growth In Culture. *J. Cell. Physiol.*, **85**, 101.

MARSHALL, C. J., FRANKS, L. M. & CARBONELL, A. W. (1977) Neoplastic Transformation in Epithelial Cell Lines Derived from Human Carcinomas. *J. natn. Cancer Inst.*, **58**, 1743.

RHIM, J. S. & HUEBNER, R. J. (1973) Transformation of Rat Embryo Cells *in vitro* by Chemical Carcinogens. *Cancer Res.*, **33**, 695.

SANFORD, K. K., HANDLEMAN, S. L., FOX, G. H., BURRIS, J. F., HURSEY, M. L., MITCHELL, J. T., JACKSON, J. L. & PARSHAD, R. (1974) Effects of Chemical Carcinogens on Neoplastic Transformation and Morphology of Cells in Culture. *J. natn. Cancer Inst.*, **53**, 1647.

STEUER, A. F., HENTOSH, P. M., DIAMOND, L. & TING, R. C. (1977) Survival Differences Exhibited by Normal and Transformed Rat Liver Epithelial Cell Lines in the Aggregate Form. *Cancer Res.*, **37**, 1864.

WAYMOUTH, C. (1959) Rapid Proliferation of Sublines of NCTC 929 (Strain L) Mouse Cells in a Simple Chemically-Defined Medium (MB 752/1). *J. natn. Cancer Inst.*, **22**, 1003.

WEINSTEIN, I. B., ORENSTEIN, J. M., GEBERT, R., KAIGHN, M. E. & STADLER, U. C. (1975*a*) Growth and Structural Properties of Epithelial Cell Cultures Established from Normal Rat Liver and Chemically Induced Hepatomas. *Cancer Res.*, **35**, 253.

WEINSTEIN, I. B., YAMAGUCHI, N., GEBERT, R., KAIGHN, M. E. & STADLER, U. C. (1975*b*) Use of Epithelial Cell Cultures for Studies on the Mechanism of Transformation by Chemical Carcinogens. *In vitro*, **11**, 130.

WEISS, R. A., VESELY, P. & SINDELAŘOVA, J. (1973) Growth Regulation and Tumour Formation of Normal and Neoplastic Rat Cells. *Int. J. Cancer*, **11**, 77.

WIGLEY, C. B. & FRANKS, L. M. (1976) Salivary Epithelial Cells in Primary Culture: Characterization of Their Growth and Functional Properties. *J. Cell Sci.*, **20**, 149.

WILLIAMS, G. M., ELLIOTT, J. M. & WEISBURGER, J. H. (1973) Carcinoma after Malignant Conversion *In vitro* of Epithelial-like Cells from Rat Liver Following Exposure to Chemical Carcinogens. *Cancer Res.*, **33**, 606.